# Farmers’ knowledge, attitudes and practices towards management of cassava pests and diseases in forest transition and Guinea savannah agro-ecological zones of Ghana

**DOI:** 10.12688/gatesopenres.13114.2

**Published:** 2021-02-18

**Authors:** Benedicta Nsiah Frimpong, Allen Oppong, Ruth Prempeh, Zipporah Appiah-Kubi, Linda A. Abrokwah, Moses B. Mochiah, Joseph N. Lamptey, Joseph Manu-Aduening, Justin Pita

**Affiliations:** 1 CSIR - Crops Research Institute, Kumasi, Ghana; 2 University of Félix Houphouët Boigny, Abidjan, Cote d'Ivoire

**Keywords:** Manihot esculenta, diseases, cassava, knowledge, perception, Ghana

## Abstract

**Background**: Cassava is a major staple root crop in Ghana, which serves as a food security and an income generating crop for farming families. In spite of its importance, the crop is plagued with biotic factors such as pests and diseases, resulting in yield and income reductions.

**Methods**: Farmers’ knowledge, attitudes and practices towards cassava pest and disease management were investigated. A mixed research questionnaire was used to collect both qualitative and quantitative data from 94 cassava farm households across two major cassava growing agro-ecologies.

**Results**: Using descriptive statistics, parametric and non-parametric analysis, our study revealed that farmers’ knowledge on cassava pests was high but low for diseases. Whiteflies ( *Bemisia* *tabaci *Gennadius), grasshoppers ( *Zonocerus variegatus*), aphids ( *Aphis gossypii *Glover), mealybugs ( *Phenacoccus manihoti*), termites ( *Isoptera*), and grasscutters ( *Thryonomys swinderianus*) were perceived as the most common damaging pests. Farmers’ descriptions showed that disease pathogens attacked foliar tissues, stem and root tissues and caused leaf dropping and die back. Cassava mosaic disease and root rot were the most common diseases; however, disease descriptions suggested the incidence of viral, bacterial and fungal diseases. Some of the farmers observed mixed symptoms on their farms. The results also showed that only 25.5% cultivated improved varieties. Management actions applied included field sanitation practices and pesticide application. The effectiveness level of the control actions was rated moderately effective.

**Conclusions**: The analysis showed heterogeneity in personal and farm level characteristics of respondents across the two agro-ecologies, but agro-ecologies were independent of the management practices employed. There is a need to improve farmers’ access to improved disease-free planting materials through efficient dissemination pathways and increase farmers’ knowledge on cassava pests, diseases and integrated management through publfic awareness creation and capacity building by extension agents and research institutions. Continued government investment is needed to achieve sustainable outcomes.

## Introduction

Global food production is expected to increase substantially by 2050 to meet the increasing demands of the burgeoning population coupled with changing diets and the growing per capita consumption that is a function of rising incomes in many countries ( Ray *et al*., 2013; Tilman *et al*., 2011). Agriculture continues to be a pillar of the population, with around 54% of the population (especially, those in rural areas) relying on it for their livelihoods. Agriculture contributed 18.3% to Ghana’s Gross Domestic Product (GDP) in 2017 ( MoFA, 2017). Crop production, especially cassava, is an important economic activity in Ghana and the world at large. It serves as food for 800 million people in the world ( Fondong & Rey, 2018). Ghana is the third leading cassava producer in Africa and with a world share of 6.3%, it ranks sixth in terms of value and volumes. Per capita consumption per year in Africa is estimated at 80 kg and 152.9 kg for Ghana ( MoFA, 2009; Shipman, 2017 cited in Boateng, 2015). Cassava contributes 46% of the country’s agricultural GDP through trade ( Sen Nag, 2017). Cassava has a broad agro-ecological adaptation and an in-ground storage capability and capacity that allows for flexible harvesting to ensure all year-round food availability ( Sanginga & Mbabu, 2015; Torkpo *et al*., 2017). The commodity once described as “food for the poor” ( FAO, 2013) is consumed in all regions of Ghana ( Bayitse *et al*., 2017) and is now envisaged as a strategic crop that could transform the Ghanaian economy as it has great potential for industrialization due to its high starch content and varied product utilization ( Bayitse *et al*., 2017). In 2017, domestic production stood at 19,137.94 metric tons, which was a 7.5% increase over the previous year’s output. This resulted from a 5.3% change in cropped area due to the government program; Planting for Food and Jobs ( MoFA, 2017). In spite of its potential, cassava’s productivity in African smallholder’s farming systems is still below the optimal level ( Elegba *et al*., 2013), though several efforts have been made by projects and programs in disseminating improved cultivars and integrated pest and disease management practices ( Rusike *et al*., 2010; Zinga *et al*., 2013).

The gap in yield has been attributed to several factors, including plant diseases that result in food scarcity ( Zadoks, 2008). Cassava, with its long life cycle, is affected by numerous diseases and pests such as *African cassava mosaic virus*, cassava bacterial blight, anthracnose, root rot, green mite and whiteflies. The most devastating within the West African belt has been the Cassava mosaic virus ( Elegba *et al*., 2013; Manu-Aduening *et al*., 2007 and Moses *et al*., 2015). Cassava diseases are reported to cause losses of fresh roots as well as planting material. Reduction in root yields could differ considerably with the cassava cultivars’ vulnerability, changes in climate and the inoculum pressure ( Fondong & Rey, 2018; Kintché *et al*., 2017). Pest and disease spread have been on the increase due to cross-border trade, movement of people from one country to another and the sharing of planting materials amongst producers and countries resulting from regional integration ( Echodu *et al*., 2019).

With the high losses linked to pests and diseases, adoption of resistant cultivars and good management strategies in Ghana have not been encouraging, ostensibly because farmers have no or little access to cultivars and little knowledge of the pests and diseases as well as associated management practices. This is evidenced in studies that have investigated this issue in Ghana ( Acheampong *et al*., 2013; Cudjoe *et al*., 2005; Manu-Aduening *et al*., 2007; Torkpo *et al*., 2017). These few studies are often skewed towards examining farmers’ knowledge and or perception with little efforts to examine attitudes and practices; thereby not wholly applying the knowledge, attitudes and practices (KAP) model to better understand farmers’ behavior and actions. Therefore, this paper is intended to fill this gap. The KAP assessment reveals what people know about the issue being investigated, how they feel and their present actions. The framework assumes that a change in practices is the cumulative result of a change in knowledge and attitudes ( Schreinemachers *et al*., 2017). Despite the vital role of farmers’ knowledge in controlling and mitigating pests and diseases, the application of the KAP model is limited or non-existent in this discipline and our study intends to enrich the body of existing literature in this area. Efforts to improve the management of pests and diseases of cassava are likely to be hindered if farmers’ knowledge on crop pests and diseases and practices for handling them are not known and considered appropriately. Thus, understanding farmers’ knowledge, attitudes and practices related to crop pests and diseases and their management is central to crop protection through identifying farmers’ training needs and is necessary for the formulation and development of effective integrated management strategies. This study was specifically designed to:

- Examine farmers’ knowledge and experience of cassava pests and diseases
- Identify farmers’ attitudes towards cassava pests and diseases
- Identify farming practices adopted in coping with cassava pests and diseases
- Examine the relationship between management practices applied by farmers and agro-ecologies
- Find out the preferred cassava variety traits of farmers.

The following two hypotheses were tested;

1. H _0_: There are no differences in the means of the quantitative variables (age, years in school, farming experience, percentage damage caused by pests and diseases, cassava farm size) of respondents across the regions.
2. H _0_: Pest and disease management practices and agro-ecological zones are independent

## Methods

### Ethical statement

Formal ethical approval was not obtained as the CSIR-Crops Research Institute has not yet put in place an ethical standing committee and according to local regulation is not required for studies not involving the collection of medical samples. The CSIR-Crops Research Institute administration approved the project and all its activities before the project was implemented. Though the Institute has not yet put in place an ethical standing committee, the project met all the institute’s expectations, guidelines and ethics. The study was conducted in line with Ghana’s Council for Scientific and Industrial Research Act, 1996 (Act 521) and the Data Protection Act, 2012 (Act 843). Moreover, respondents were informed about the purpose of the study and use of their responses in a language they understood and willingly gave their consent prior to data collection. Because participants could not read and write, informed consent was sought verbally, which was recorded via audio taping, as approved by the CSIR-Crops Research Institute. The data being presented do not include any personal data via which a specific respondent could be identified or traced.

### Description of study areas

The study was conducted in two cassava agro-ecologies of Ghana, Guinea Savannah and Forest Transition, involving two study districts. These two agro-ecologies were originally part of regions selected for a nationwide disease surveillance in 2015/2016/2017 as part of the implementation of the West Africa Virus Epidemiology Project funded by the Bill and Melinda Gates Foundation and the Department for International Development, UK. The chosen agro-ecologies are part of the major cassava growing agro-ecologies in the country. The Eastern region which is within the Forest Transition is ranked 1 ^st^ and the Northern region which falls within the Guinea Savannah ranks 5 ^th^. The former region recorded a production level of 4,649,507.58 tons and the latter of 1,428,427.53 tons in 2016 ( MoFA, 2016). With a total area of 70,384 km ^2^, the Northern region is the largest region in terms of land mass. The total population was estimated at 2,479,461 in 2010 with 50.4% females (1,249,574) and 49.6% males (1,229,887). More than 75% of the economically active population depends on agriculture for survival. The region has a relatively dry climate with a single rainy season beginning in May and ending in October, with rainfall distribution varying between 750 mm and 1,050 mm. With the harsh climate experienced in this region, cassava serves as a resilient and secure food commodity for the populace ( Ghana Statistical Service, 2013).

**Figure 1.**
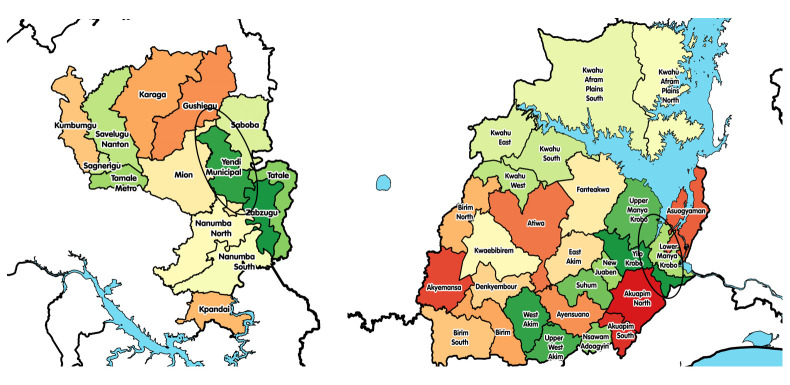
District Map of Northern and Eastern Regions of Ghana with study areas in the circle. Maps were adapted from original versions available from Wikimedia under a CC BY-SA 4.0 license (Northern region map – Rwhaun, 2018, https://commons.wikimedia.org/wiki/File:Districts_of_the_Northern_Region_(2018).png; Eastern region map – Macabe5387, 2017, https://commons.wikimedia.org/wiki/File:Districts_of_the_Eastern_Region_(2012).svg).

The Eastern region covers a total land mass of 19,323 km ^2^ with an estimated population of 2,633,154 in 2010 based on the Population and Housing Census (PHC). 51% are females (1,342,615) and 49% males (1,290,539). The vegetation is tropical with two seasons; dry and wet. The climate and soils support a variety of cash and food crops including cocoa, kola, cassava, rice and oil palm, which account for 70–85% of agricultural output. Cassava dominates in terms of cropped area and total production ( MoFA, 2011) and the region ranks 1 ^st^ in cassava production ( MoFA, 2016). In this region, 53% are employed in the agriculture sector ( Ghana Statistical Service, 2013). The study districts fall within the region’s major cassava districts (as shown on the Ministry of Food & Agriculture (MoFA) website and in the map in Figure 1).

### Sample size and sampling technique

A two-stage sampling technique was used to select the study districts and sample. The two districts, Yendi Municipal in the Guinea savannah region and Lower Manya Krobo in the forest transition region were purposively selected due to the importance of cassava in these districts and as part of the disease surveillance survey districts. A simple random sampling technique was used to select 10 cassava farmers each from five randomly selected communities within each district, resulting in a total of 100 respondents. Preliminary engagements were made with the district directors of agriculture to inform them of the purpose of the study but not with the respondents. The farmers were drawn from a list obtained from the Root and Tuber Improvement and Marketing Programme (RTIMP) desk officer at the district department of agriculture. The list was inputted into Excel and random numbers were generated to assign to respondents using the RAND function. The INDEX and RANK functions in excel were used to select the 100 participants. The participants selected for a face-to-face interview were informed through district directors of agriculture. Due to incompleteness of some of the questionnaires, 94 valid responses were retrieved, representing a 94% response rate.

### Data collection

Data collected was mainly primary and was accomplished in 2017. Data was collected using a semi-structured interview guide ( Nsiah Frimpong *et al.*, 2020b) administered to the sampled respondents by interdisciplinary researchers (social scientist: BNF, pathologists: AO, ZAK, LA, JP and breeders: RP, JMA) with MSc and PhD qualifications and trained enumerators with Diplomas and BSc degrees in Agriculture (both males and females) from the districts’ departments of agriculture. The interviews took place at the homes of the respondents, either in the presence of other family members or individually and lasted for a maximum of 45 minutes. Responses were either ticked or transcribed as and where appropriate. A mixed research questionnaire was adopted with both closed and open-ended questions, which elicited both qualitative and quantitative research variables. This integration was deemed appropriate as it provided an in-depth understanding of the questions being posed in the survey. The survey instrument was designed in English and translated to the local language by the trained enumerators during field administration. Prior to field data collection, the research team and trained enumerators pretested the survey instrument to ensure consistency and logical flow of questions.

Individual cassava farmers from selected households were chosen as the unit of analysis and data collected covered their demographics, farm level characteristics, knowledge and experiences with cassava pests and diseases, management practices employed and their effectiveness, sources of information on pests and diseases, knowledge on improved varieties and preferred attributes, willingness to cultivate improved varieties and knowledge of the new devastating cassava disease caused by ‘cassava brown streak virus’. There were several triangulations to check and confirm farmers’ responses on experiences with practices of managing cassava pests and diseases, variety names, characteristics and categorization. Triangulation involves using different methods to collect data on same topic to ensure validity of results. There are four types of triangulation; data, investigator, theory and methodological ( DźwigoŁ, 2018) and the study used mostly investigator triangulation. For instance, to confirm respondent’s knowledge on pests and diseases, he/she was asked to name and describe the associated symptoms. Within the multi-disciplinary team, researchers relied on the viewpoints of the subject matter experts to validate pest and disease names/descriptions and variety names, attributes and categorization into traditional and improved during analysis. 

At the beginning of the interview, enumerators explained the purpose of the study and content of the questionnaire to the respondents and assured them of the confidentiality of the data collected. Verbal consent to participate in the study was sought from all respondents explicitly in their mother tongue before proceeding to start the interview. Participants could freely terminate the interview at any time if they felt uncomfortable with some of the questions. During the interview, questions regarding pest and disease encounter and management were repeated and further explained to some of the farmers for clarity and to elicit correct responses. Farmers’ responses were constantly read to them for confirmation. Once the responses were read to them, everything was clarified in the field. However, farmers’ contact details were taken in order to make a follow up phone call to clarify issues should the need arise during analysis, but no such call back was done or feedback on findings given.

### Data analysis

Data collected was inputted using SPSS 20 software and were analyzed using descriptive and inferential statistics. The descriptive statistics included frequencies, cross tabulations, charts, means, minimums, maximums and standard deviations. The study employed both parametric and non-parametric tests, which included the t-test and Chi-square test (Fisher’s exact). The Chi-square test of independence was first run to test whether any relationship existed between the agro-ecologies and the management measures employed; however, because the expected cell values were less than five, which violated the Chi-square assumption, researchers resorted to the Fisher’s exact test, which qualified for that purpose. Farmers’ attitudes were linked to behavioral control methods such as variety cultivated, awareness of and willingness to cultivate improved varieties, cropping system and choice of planting dates. Practices referred to the actions taken or coping strategies used when they encountered issues with pests and diseases. The effectiveness level of the actions was evaluated on a three-point scale; 1 = “not effective”, 2 = “moderately effective” and 3 = “very effective”. Inter-regional comparisons were carried out to ascertain heterogeneity in the quantitative variables measured using the t-test. These helped to draw conclusions about the population from which the sample was selected. Open-ended questions were coded by reading through the transcripts severally and themes built for textual analysis. Coding was manually done by two of the interviewers (BNF and RP) and perspective of other experts in the team sought to aid in the organization of responses. For instance, codes like “mounds”, “slanting”, “cut stem/sticks” were developed for how the planting of cassava is done and “curled leaves”, “rotten roots”, “yellowing”, “chew leaves”, “white pests”, were developed for knowledge on and experience with pests and disease. At the point where researchers realized they had no more codes or new information, the codes were developed into themes. The textual analysis involved examining the text and analyzing the sentence structure by combining both content and narrative analysis for the open-ended responses. While the content analysis dealt with categorizing responses under and into themes, narrative analysis looked at evaluating farmers’ descriptions for pests, diseases, planting methods, varietal characteristics.

## Results

### Respondent demographics

A total of 94 respondents were selected for the study; approximately 53% from the Guinea savannah agro-ecology and 47% from the forest transition zone ( Table 1). Table 1 shows that 77.7% of the respondents were males and 22.3% females. The majority of the respondents (69.1%) have had some formal education taken at different levels (basic, secondary and tertiary), with many of them clustering around the basic level. A higher percent (91.5%) were married, which presupposes the support farmers could get from their partners. Again, the majority (74.5%) of the respondents were household heads and 77.7% were members of an agricultural organization. Descriptively, the analysis revealed some variations across locations in relation to the categorical variables; level of education, respondents being head of household and membership of agricultural organization, as presented in Table 1.

**Table 1.** Demographic characteristics (categorical variables).

Variables	All		Guinea savannah		Forest transition	
	No.	%	No.	%	No.	%
**Number of** **respondents by** **agro-ecology**	**94**	**100**	**50**	**53.2**	**44**	**46.8**
**Sex** Male Female	73 21	77.7 22.3	37 13	74.0 26.0	36 8	81.8 18.2
**Educational level** None Basic Secondary Tertiary	29 36 18 11	30.9 38.3 19.1 11.7	22 14 10 4	44.0 28.0 20.0 8.0	7 22 8 7	15.9 50.0 18.2 15.9
**Marital status** Single Married Divorced Widowed	5 85 2 1	5.3 91.5 2.1 1.1	2 47 1 0	4.0 94.0 2.0 0	3 39 1 1	6.8 88.6 2.3 2.3
**Head of household** Yes No	70 24	74.5 25.5	33 17	66.0 34.0	37 6	86.0 14.0
**Membership of an** **agricultural** **organization** Yes No	73 21	77.7 22.3	41 9	82.0 18.0	32 12	72.7 27.3

### Test of equality in means between respondents from the two regions

The independent sample t-test was conducted to test for equality in the means of characteristics of respondents in the two study districts. The null hypothesis stated that the means of quantitative variables of respondents from the two agro-ecologies are the same. The F-statistic and p-values of the Levene’s test of equality of variance showed that the test assumes equal variance for all the variables except years in school, experience in cassava farming and household size, which had p<0.05. Details of the Levene’s test results are presented as *Extended data* ( Nsiah Frimpong *et al.*, 2020b). The results showed some differences across the agro-ecologies. Differences in age, years of cultivating cassava, years in school and household size were significant at 1%, while farming experience and proportion of farm damaged by diseases were significant at 5% and the number of adult males actively involved in farming was significant at 10% ( Table 2).

**Table 2.** Independent sample t-test results (continuous variables).

Variables	Guinea savannah		Forest transition		P-value
	Mean	Std. Error	Mean	Std. Error	
Age	39.48	1.672	48.23	1.793	0.001 ***
Farming experience	16.76	1.384	21.95	1.629	0.016 **
Years of cultivating cassava	11.98	1.155	17.89	1.701	0.004 ***
Years in school	6.02	0.834	9.11	0.739	0.007 ***
Household size	11.04	0.598	7.23	0.469	0.000 ***
Household members actively involved in farming	4.26	0.403	5.0	0.464	0.229
Adult males involved in farming	1.42	0.181	1.93	0.201	0.061 *
Cassava farm size (hectares)	2.12	0.253	2.43	0.274	0.409
Percentage of farm damaged by pest	27.94	3.764	19.97	3.773	0.153
Percentage of farm damaged by diseases	25.91	2.981	15.53	3.079	0.018 **
Number of extension contacts	9.06	0.348	8.81	0.371	0.622

***, **, and * represent 1%, 5% and 10% significance levels, respectively.

### Farmers knowledge of cassava pests and diseases

In ascertaining farmers’ knowledge, three aspects were assessed; experience with any pests and or diseases, name of pests and diseases and description of pests and diseases encountered. Results in Table 3 show the regional trend, indicating that majority of the farmers, 74% and 70.5% from Guinea Savannah and Eastern regions, respectively, experienced pests on their farms, while 72.0% and 70.4% farmers, respectively, experienced diseases, with the last period of encounter being quite recent (2014–2017). It was found that 61.8% of the farmers perceived whiteflies ( *Bemisia tabaci* Gennadius), grasshoppers ( *Zonocerus variegatus*), aphids ( *Aphis gossypii* Glover), mealybugs ( *Phenacoccus manihoti*), termites ( *Isoptera*), and grasscutters ( *Thryonomys swinderianus*) as the most common and damaging pests to their crops. The remaining 38.2% could not give specific names but generally described them as “whitish insects”, “flies” or with symptoms such as “create holes or chew leaves” (Questionnaire ID 11 & 12), “web/wax-like patterns on the leaves” (Questionnaire ID 17), “pests clustering beneath the leaves and causing wrinkles” (Questionnaire ID 41), “yellowing of leaves and leaves dropping” ( Nsiah Frimpong *et al.*, 2020a). More farmers in the Guinea savannah region were able to give specific names of the pests than those from the Forest transition region.

**Table 3.** Farmers’ knowledge of cassava pests and diseases.

Variables	All		Guinea savannah		Forest transition	
	No.	%	No.	%	No.	%
**Experience harmful pests** Yes No	68 26	72.3 27.7	37 13	74.0 26.0	31 13	70.5 29.5
**Experience diseases** Yes No	67 27	71.3 28.7	36 14	72.0 28.0	31 13	70.4 29.5
**Names of common pests** Whiteflies Grasshoppers Aphids Mealybugs Termites Grasscutters	29 9 8 3 2 1	47.5 14.8 13.1 4.9 3.3 1.6	14 6 7 2 2 0	38.9 16.7 19.4 5.6 5.6 0	15 3 1 1 0 1	60.0 12.0 4.0 4.0 0.0 4.0
**Names of common** **diseases** Cassava mosaic disease Root rot	15 13	22.4 19.4	11 4	30.6 11.1	4 9	12.9 29.0

The predominant diseases known by the farmers were the cassava mosaic disease (22.4%) and root rot (19.4%), which are viral and fungal diseases, respectively. The majority (58.2%) of the farmers could not give specific names but used descriptions such as “black/brown leaf spots”, “curled leaves”, “mottled leaves”, “yellowing of leaves”, “leaves dropping and stunted growth” to demonstrate their knowledge of diseases. Out of the 67 farmers who experienced diseases, only 28 farmers, representing less than half (41.8%), could explicitly give the names of the diseases that attacked their farm. Cassava mosaic disease was experienced more in the North and root rot was experienced more in the Eastern region. The mean damage caused by pests and diseases was 22.84% and 21.02%, respectively, and ranged from 0–100%. Results on the distribution and peak of percentage damage by pests and diseases data showed that pest damage data is slightly skewed and flat, while disease damage data showed normal distribution and flatness; hence not many issues with skewness and kurtosis in the data ( Table 5). Out of the 67 respondents who experienced cassava diseases, 74.6% reported single infections and 25.4% observed multiple symptoms. Farmers’ responses on knowledge and experiences of cassava diseases were validated by triangulation using seven statements that described the common symptoms of bacterial, fungal and viral cassava diseases ( Table 4). With these prompts, five more respondents confirmed the observance of symptoms on their farm. Multiple symptoms were observed by 21.3% of the farmers; hence the probability of multiple disease incidence. In addition to the cassava mosaic disease and root rot reported by farmers, the symptoms statements revealed that brown leaf spot (35.1%) and white leaf spot (22.3%) diseases could also be common in the districts.

**Table 4.** Farmers Response to Disease-symptom statements.

Disease symptoms	Percentage of farmers affected.
Brown spots on the leaves	35.1
White spots on the leaves	22.3
Whitish substance/web-like strains on the leaves	16.0
Scars on cassava stems/nodes	16.0
Leaf distortion, stunting or yellowing of leaves	42.6
Progressive death of the leaves and other parts of the plant starting from the tips	19.1
Rot in the tubers after harvest	28.7

**Table 5.** Distribution of pests and disease damage.

Variable	Minimum	Maximum	Mean	Standard deviation	Skewness	Kurtosis
Damage by pests	0	100	22.84	20.94	1.22 (0.291)	1.33 (0.574)
Damage by diseases	0	70	21.02	17.91	0.95 (0.293)	0.19 (0.578)

***Figures in parentheses represent standard errors***

### Farmers’ attitudes and practices in cassava pests and diseases management

**Figure 2.**
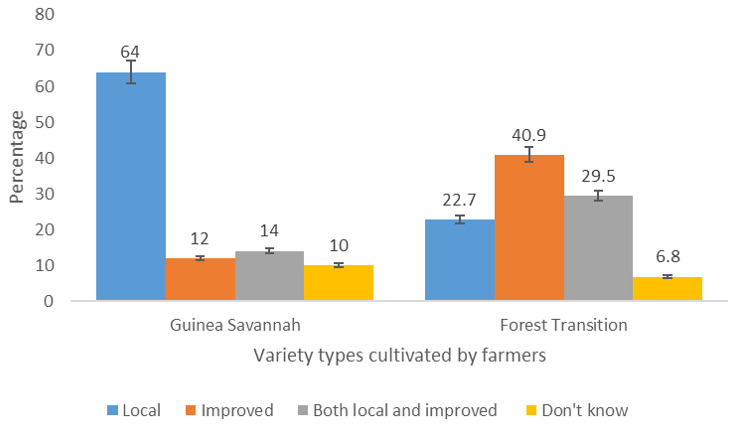
Graph of variety types cultivated by farmers.

The majority of farmers (74.5%) intercropped cassava with cereals (maize, sorghum and millet), other roots and tubers (yam and cocoyam), legumes (groundnut, cowpea, pigeon pea and soybean) and vegetables (pepper, okro, tomatoes and garden eggs). Maize (67.1%) and yam (42.9%) were the most common ( Table 6). The variety names were verified by technical experts in the team and classified into local or improved varieties; 64% of farmers in the Guinea savannah region cultivated only landraces compared with 22.7% in the forest transition region. Solely improved varieties were cultivated by 40.9% farmers in the forest transition region and 12% in the Guinea savannah region ( Figure 2). Table 7 shows the list of local and improved varieties identified in the area. Though the majority (86.2%) of the farmers were aware of improved varieties ( Table 8), the local landraces dominated ( Figure 2).

**Table 6.** Distribution of cassava cropping system.

Variables	Frequency	Percentage
**Cassava cropping** **system**		
Sole crop	24	25.5
Intercrop	70	74.5
**Main crops** **associated with** **cassava**		
Maize	47	67.1
Yam	30	42.9

**Table 7.** Names of varieties cultivated in the study areas.

Local varieties	Improved varieties
Gbedze	Ankra
Kumasi	Filindiakong
Osangmonor/ Lagos/ Nigeria	Afisiafi
Tuaka	Bankyehemaa
Techiman	Sika Bankye
Bosomensia	Nyerikobga
Yoribawa	Ampong
Sanyoo	
Navrongo	
Lucas	
Drogbo	
Accra boy	
Dowasla	

**Table 8.** Distribution of farmers’ awareness and willingness to cultivate improved varieties.

Variables	Frequency	Percentage
Awareness Yes No	81 13	86.2 13.8
Willingness Yes	94	100

Most farmers planted cassava around March/April/ May. More than 90% of the farmers planted the cassava by cutting the sticks into pieces and slanting it on mounds. The remainder planted on mounds but in a vertical position. Harvesting was normally done from 6–24 months after planting depending on the variety cultivated.

 All (100%) farmers were willing to cultivate improved varieties, locally referred to as “ *agric bankye*”, provided they could access planting materials ( Table 9). Qualitatively, the top five desired traits/characteristics included high yielding (big tubers), early maturing (6 months to 1 year), multiple uses/by-products *(“gari”, “fufu” and starch*), disease resistance and tolerance to harsh weather conditions. There was slight variation in the traits preferred by male and female farmers. Most of the males preferred high yielding (big tubers) and early maturing varieties, but the females preferred early maturing, multiple food uses and disease resistant varieties.

**Table 9.** Distribution of immediate action taken by farmers in pest and disease management.

Variables	Pest infestation (n=94)		Disease infection (n=94)	
	Immediate Percentage	Long-term Percentage	Frequency Percentage	Long-term Percentage
Reported to agricultural officer	45.6	5.9	43.3	3.0
Field sanitation	19.1	30.8	22.4	43.3
No action taken/ control	26.5	33.8	32.8	37.3
Pesticide application	8.8	29.4	1.5	16.4

Farmers’ behavior in coping with pests and diseases revealed four options. Similarities existed in both farmers’ immediate and long-term responses. Table 9 indicates that 45.6% immediately contacted an agricultural extension officer, 19.1% employed field sanitation/cultural practices, 26.5% took no action and 8.8% applied pesticides. As long-term measures, 33.8% took no further action, 29.4% sprayed chemicals, 30.8% observed cultural practices with some extension advice and 5.9% invited extension officers to the scene ( Table 9). Similar management strategies were employed for diseases. Questions on the effectiveness of pest management strategies indicated that 17.6% believed the practices resorted to were very effective, 36.8% believed they were moderately effective and 45.6% indicated they were ineffective. With a mean score of 2.3 and standard deviation of 0.750, the overall level of effectiveness of the pest management measures was perceived as being moderately effective. With disease management, 20.9% perceived the practices as being very effective, 38.8% as moderately effective and 40.3% as ineffective, with an overall mean score of 1.8 ( Table 10).

**Table 10.** Rating of the level of effectiveness of pests and disease management practices.

Variables	Pooled sample		Mean score	Standard deviation
	No.	%		
**Effectiveness of pest** **management measure**				
Very effective Moderately effective Not effective	12 25 31	17.6 36.8 45.6	2.3	0.750
**Effectiveness of disease** **management measure** Very effective Moderately effective Not effective	14 26 27	20.9 38.8 40.3	1.8	0.764

### Testing the relationship between agro-ecologies and farmers’ pest and disease management practices

A more robust test such as the Fisher’s exact test was appropriate to correct for the small frequency values in each cell and to satisfy the assumption of carrying out a Pearson Chi-Square test. The results in Table 11 and Table 12 show that since the P-values (0.357) and (0.697) were greater than 0.05 for pest and disease management practices, we do not reject the null hypothesis of “no association between agro-ecology and choice of practices”. This means that the two categorical variables are not related and thus, agro-ecologies are independent of the choice of pest and disease management practices.

**Table 11.** Fisher’s exact test for cassava pest management practices by agro-ecology.

Agro-ecology	Actions taken by farmers to manage cassava pests			
	Report to agricultural officer	Field sanitation	No action taken	Pesticide application
Guinea savannah	3 (8.1)	11 (29.7)	15 (40.5)	8 (21.6)
Forest transition	1 (3.2)	10 (32.3)	8 (25.8)	12 (38.7)
Fisher’s exact test (P-value: 0.357>0.05)				

***Figures in parentheses represent percentages***

**Table 12.** Fisher’s exact test for cassava disease management practices by agro-ecology.

Region	Actions taken by farmers to manage cassava diseases			
	Report to agricultural officer	Field sanitation	No action taken	Pesticide application
Guinea savannah	1 (2.8)	17 (47.2)	14 (38.9)	4 (11.1)
Forest transition	1 (3.2)	12 (38.7)	11 (35.5)	7 (22.6)
Fisher’s exact test (P-value: 0.697>0.05)				

***Figures in parentheses represent percentages***

### Sources of information on cassava pests and diseases

**Figure 3.**
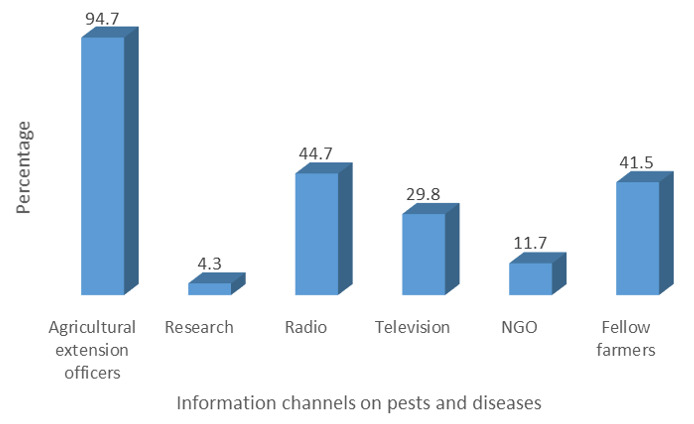
Information channels on crop pests and diseases. NGO, non-governmental organization.

Figure 3 indicates that the majority of farmers (94.7%) depended on the extension directorate under MoFA for information on pests and diseases, with the least amount of information sourced from research institutions. Other sources included radio (44.7%), fellow farmers (41.5), television (29.8%) and non-governmental organizations (11.7%).

## Discussion

The purpose of the study was to examine and understand farmers’ knowledge, attitudes and practices in coping with cassava pests and diseases. Insights from this is central to crop protection and necessary for the development of effective integrated management strategies. The farmers’ characteristics revealed that most of the farmers have had some level of formal education and the study observed an improvement over other studies ( Asare-Bediako, 2014), which have reported low levels of education among farmers in rural areas. This observation may be due to the introduction of the Free and Compulsory Universal Education (FCUBE) policy introduced in Ghana in 1995 ( Akyeampong, 2009), which gave most of the people in rural Ghana, especially Northern Ghana, the opportunity to access education ( Ghana Statistical Service, 2013). The moderate level of education of farmers could expedite technology uptake and impact positively on any developmental program executed in the regions. The high number of farmers being household heads is a promising indicator as it could stimulate quick on-farm decision making in the area of investment and resource allocation, since the household heads control family resources. Membership of agriculture organizations presents an avenue to receive current information on agriculture and, more importantly, on pests and diseases, as well as support for advancing the farm as projects prefer working with organized groups.

According to Bloom’s taxonomy as cited in Kusumawardani *et al.* (2019), knowledge is the primary step of perception, which then generates attitudes and results in actions. With farmers representing a broad demographic pool with regard to experience, educational level, age, household head and household size, the study observed that the majority of the farmers had knowledge of the damaging pests specific to their crop. This finding is consistent with Echodu *et al.* (2019). The pests mentioned by farmers were among some of the common devastating cassava pests well documented in literature ( PlantVillage and Mathieu-Colas *et al*., 2009). Farmers’ over-reliance on their own experiences, intuition and farmer-to-farmer networks for knowledge of different pests could be problematic as the right information may be missed. Farmers were unaware of specific insect vectors with multiple species like whiteflies and mealybugs and classified them under one generic name, which could affect the appropriate management strategy being employed. Cudjoe *et al.* (2005) made a similar observation and reported that farmers were generally unaware of the *Bemisia* whiteflies and could not identify them by specific local names. Most of the pests mentioned by farmers are known to be insect vectors of viral, bacterial and fungal cassava diseases ( Fanou *et al*., 2017). For instance, whiteflies cause cassava mosaic disease and grasshoppers are known to carry the pathogen that causes cassava bacterial blight ( Fanou *et al*., 2017). Farmers’ knowledge on diseases was found to be limited, which is consistent with a study by Echodu *et al.* (2019) but contrasts that of Appiah-Kubi *et al.* (2015). Some farmers, though they noticed changes in the plant tissues, were unable to associate them with specific diseases. Disease descriptions depicted that pathogens attacked foliar tissues, stem and root tissues; thus, causing leaf spots, leaf dropping, wilting, die-back and rot. Pests, diseases and related symptoms were used interchangeably, demonstrating farmers’ knowledge gap in cassava diseases. These twists in responses revealed that farmers need intensive training on simple skills and tools in pest and disease identification and management. Our study has shown that mixed infections could occur on the farm, as reported by the farmers and confirmed by the disease symptoms statements. This adds to existing knowledge, since most studies on roots and tuber diseases in Africa ( Adam *et al*., 2015; Houngue *et al*., 2018; Torkpo *et al*., 2017 and Okonya *et al*., 2019) have only reported single infections, but in an open environment it is possible for multiple infections to occur. Questions on pests, diseases and symptoms did not plunge deeper to delineate the crop cycle stage attacked and the season of the cropping year in which these pests and diseases were prominent to affirm farmers’ responses. Further studies should incorporate these stages of the crop cycle and seasons as a means of validating farmers’ responses. Manu-Aduening *et al.* (2007) in their study on *“Farmers’ perceptions and knowledge of cassava pests and diseases and their approach to germplasm selection for resistance in Ghana”* realized inconsistencies in farmers’ responses and found cassava mosaic disease, anthracnose and brown leaf spot to be present on farmers’ fields. This indicates that these diseases have persisted for a long time, and efforts and investments should be geared towards its control to avoid them becoming epidemic.

Cassava, due to its long growth cycle, is exposed to varying pests, diseases and climatic conditions and prevention of pests and diseases requires an integrated approach. Integrated pest and disease management practices have been developed by research institutions but on balance, adoption by farmers is low ( Litsinger *et al.*, 2009), either due to farmers’ inadequate knowledge on these practices or lack of skill to implement them. In this study, a little over a third of the respondents “did nothing” in the long run to manage pests and diseases. Our findings were consistent with similar studies by Torkpo *et al.* (2017) in Ghana and Gbaguidi *et al*. (2005) in Benin, who found that farmers hardly adopted the management strategies that prevented the spread of diseases. Farmers opined that they did not know what to do and others, even after reporting to the extension officers, received no help. It could be deduced that extension services and training support for pest and disease management are inadequate. It is worth mentioning that some farmers, based on their own experiences or advice from extension agents, employed some cultural practices such as killing the pests by hand, rouging, burning infested plants and weed management to improve crop vigor. This is consistent with Ntonifor *et al*. (2005) in Cameroon, who also found that nearly 30% of the farmers practiced rouging and other management practices. Nevertheless, only one person (1.1%) practiced crop rotation and fallowing. These are practices that have been promoted for a long while, but their application is still low, and this demonstrates farmers’ attitudes towards pest and disease management. So, as a long-term solution, some farmers resorted to pesticide application, which is expensive and could cause health hazards. Biological control was virtually nil (1.1%) as a single respondent sprayed boiled neem leaves, which was seen as effective. The neem tree ( *Azadirachta indica*), which is a common tree in the tropics including in Ghana, has been promoted by both agro-foresters and pathologists because of its multiple uses ( Saxena, 2015). Since this is common in the Ghanaian environment, it must be promoted among our smallholder farmers to sustainably manage pests and preserve the environment and biodiversity. We can conclude from the findings that integrated pest and disease management strategies are rarely implemented and farmers that implemented some practices were unaware of the benefits and thus may not do it diligently. Similar observations were made by Gbaguidi *et al*. (2005) in Benin and Torkpo *et al.* (2017) in Ghana. Based on these revelations, there is a need for strong partnership and all stakeholders must join in the fight against disease and pest attacks. Research institutions and the directorate of extension under MoFA still have much work to do in assisting farmers manage and control pests and diseases. Research should regularly build capacity of extension agents, develop packages of integrated pest and disease management strategies in simple, concise language and in local dialect that could easily be assimilated and used by farmers.

Studies have recommended integrated management measures such as cropping systems (intercropping with non-host plants), cultivation of improved resistant varieties, cultural practices (removal and destroying infested plants, crop rotation, fallowing, proper weed management), management of planting dates based on agro-ecology, and soil amendments, which usually results in high yields ( Afrane Okese, 2016; Alam *et al*., 2016; Dormon *et al*., 2007; Mathieu-Colas *et al*., 2009; and Fanou *et al*., 2017). Intercropping has been observed as a common practice among farmers ( Appiah-Kubi *et al*., 2015; Torkpo *et al*., 2017). Farmers applied this only as a diversification strategy to reduce production risk and were unaware of its disease management capacity. Cassava intercropping is done with short duration food crops like cereals and legumes, which offers a lot of advantages. Roots and tubers like yam and cocoyam are also intercropped with cassava ( Appiah-Kubi *et al*., 2015; Torkpo *et al*., 2017). With cassava being a heavy feeder ( Mathieu-Colas *et al*., 2009 and Biratu *et al*., 2019), intercropping with leguminous crops improves soil properties as crop residue serves as mulch. Cassava in association with yam demonstrates the symbiotic relationship between the two crops. The cassava serves as stakes for the yam, while farmers believe that the cassava bulks well in the yam mounds. Intercropping, on the other hand, could have negative effects on disease spread if done with alternative host plants of certain diseases ( Appiah-Kubi *et al*., 2015). For instance, yam could host fungus that causes cassava anthracnose ( Afrane Okese, 2016). Thus, it was not surprising that some farmers observed bud necrosis on their farms.

Planting improved pest and disease resistant varieties is another way of curtailing pests and diseases. With cassava mosaic disease being the most devastating in Africa ( Elegba *et al*., 2013), improved varieties are normally screened by breeders against this disease ( MyFarm Blog). All the improved varieties cultivated by the farmers are known to be resistant/tolerant to the cassava mosaic virus; however, only 25.5% of farmers cultivated only improved varieties (seven varieties identified out of the 26 released varieties). Though there is more room for improvement, this is higher than the 11% reported by Cudjoe *et al.* (2005). Most of the local varieties, though perceived to be high yielding with multiple uses, are susceptible to some of the cassava diseases and farmers should desist from their continuous cultivation. For instance, “Tuaka” can yield 32.3 tons per hectare but is susceptible to root rot and cassava mosaic virus ( Amenu, 2015).

The study observed that pests and diseases were not farmers’ top priority in choosing a new cultivar. Pest and disease resistance ranked fourth on farmers’ preferred traits. This demonstrates that though pests and diseases are discussed as a serious matter, farmers still do not consider it as such due to lack of knowledge on the extent of both physical and economic damage caused. Farmers need to be provided with empirical evidence of the effects of pests and diseases on their livelihoods to reorient their thinking. Despite the reforms, Ghana’s seed sector is passing new legislation, which seeks to increase availability of improved varieties that are not currently readily available for farmers to access. Failure in the seed supply system in Ghana has been attributed to governance challenges that affected all stages of the supply chain ( Adu-Gyamfi *et al*., 2018). Adu-Gyamfi *et al.* (2018) reported challenges such as limited involvement of smallholder farmers in setting breeding objectives, restricted private sector participation in seed production, under-resourced public regulatory bodies to ensure proper certification and over-reliance on weak public extension systems to disseminate improved varieties. Government policies should focus on the need to address these challenges for an effective seed system in the country. 

When farmers were asked about the effectiveness of the pest and disease management strategies used, the responses showed that they were barely effective. It is evident that farmers crave more effective and efficient pest and disease management strategies. Farmers must be trained on integrated approaches to pest and disease management. This could be done by the research and extension directorate of MoFA. Information on this could also be disseminated to farmers in their local language through both print and electronic media in the form of fliers. Other channels that could be used to educate farmers, especially in the rural communities, are FM radio stations, social media (Whatsapp, Facebook, etc.) and televisions in addition to other regular sources of information.

## Conclusion and recommendations

In summary, the findings articulate the importance and prevalence of pests and diseases in the study regions, with high number of farmers affected. The study reveals good knowledge of pests but limited knowledge on diseases. The study has shown that mixed disease infections could occur, which are rarely reported in disease studies. However, integrated pest and disease management strategies were rarely applied due to a lack of knowledge and the seriousness farmers attached to pest and disease resistance traits of crops. Biological control was also rarely an option for pest and disease management. Supply of improved varieties and disease-free planting materials was still a challenge, which resulted in farmers depending on their local unimproved varieties. Although some farmers practiced intercropping, cultivated improved varieties and chose specific planting dates, they were unaware that these could have a direct correlation with pest and disease reduction on their farms.

Due to the movement of people and cross-border trade, there is the need for a multi-disciplinary collaboration within the sub-region to develop multiple resistant varieties and broad-spectrum pest and disease strategies to tackle the wide range of pests and diseases within the sub-region. The policy implication of our study is the need to improve farmers’ access to crops with improved desired traits and disease-free planting material through efficient dissemination pathways. Increased farmer knowledge on integrated cassava pest and disease management through public awareness creation and capacity building by extension agents and research institutions is also highly recommended. This measure will reduce pest and disease pressure on farms, leading to high productivity, enhanced food security, and increased incomes, among other benefits. From the research perspective, the study identified the need for the breeding team to make farmers an integral part of the breeding process, in terms of design and setting breeding priorities, to enhance uptake of improved varieties and appropriate management techniques. An empirical study using experimental tools is needed to validate yield losses reported by farmers.

## Data availability

### Underlying data

Harvard Dataverse: Farmers knowledge of cassava pests and diseases. https://doi.org/10.7910/DVN/27YW9V ( Nsiah Frimpong *et al.*, 2020a)

### Extended data

Harvard Dataverse: WAVE Knowledge Paper_Extended data. https://doi.org/10.7910/DVN/AJZDFN ( Nsiah Frimpong *et al.*, 2020b)

This project contains the following extended data:

- WAVE Knowledge Paper_Frimpong et al Wave questionnaire.doc (copy of study questionnaire)
- WAVE Knowledge Paper_ Frimpong et al Appendix.doc (details of the Levene’s test results)

Data are available under the terms of the Creative Commons Zero “No rights reserved” data waiver (CC0 1.0 Public domain dedication).
