# Supplementary material for: Farmers’ knowledge, attitudes and practices towards management of cassava pests and diseases in forest transition and Guinea savannah agro-ecological zones of Ghana
Source: Gates Open Res. 2021 Feb 18;4:101. Originally published 2020 Jul 8. [Version 2] doi: 10.12688/gatesopenres.13114.2 (PMC8012878; doi:10.12688/gatesopenres.13114.2)
Supplement: Peer review history

# Peer review: Reviewer response for version 2

**DOI:** 10.21956/gatesopenres.14453.r30424

**Published:** 2021-03-31

**Authors:** Peace Musiimenta, Losira Nasirumbi Sanya

**Affiliations:** 1 School of Women and Gender Studies, College of Humanities and Social Sciences, Makerere University, Kampala, Uganda; 2 College of Agricultural and Environmental Studies, Makerere University, Kampala, Uganda

Thank you for the opportunity to review this article. We have read through and hereby approve the paper as is.

Is the work clearly and accurately presented and does it cite the current literature?

Yes

If applicable, is the statistical analysis and its interpretation appropriate?

Yes

Are all the source data underlying the results available to ensure full reproducibility?

Partly

Is the study design appropriate and is the work technically sound?

Yes

Are the conclusions drawn adequately supported by the results?

Yes

Are sufficient details of methods and analysis provided to allow replication by others?

Partly

Reviewer Expertise:

NA

We confirm that we have read this submission and believe that we have an appropriate level of expertise to confirm that it is of an acceptable scientific standard.

# Peer review: Reviewer response for version 1

**DOI:** 10.21956/gatesopenres.14290.r29064

**Published:** 2020-08-03

**Authors:** Peace Musiimenta, Losira Nasirumbi Sanya

**Affiliations:** 1 School of Women and Gender Studies, College of Humanities and Social Sciences, Makerere University, Kampala, Uganda; 2 College of Agricultural and Environmental Studies, Makerere University, Kampala, Uganda

**Reviewed by:**

 Musiimenta, Peace, PhD and Losira-Nasirumbi Sanya, PhD

**Abstract:**

 The abstract presents the essential background that clearly depicts cassava as a major staple root crop in Ghana with potential to buttressing food security and household income generation despite is susceptibility to pests and diseases. Methods: Farmers’ knowledge, attitudes and practices towards cassava. The abstract further presents a snapshot of the methodological process, results, conclusion and recommendations.

**The team composition**:

 There is evidence that the study was conducted by a multi-disciplinary team which is commendable. The authors of the article are well established in the field of agriculture and development with requisite knowledge and experience to inform the intended audience in their discipline and policy.

**Relevance of** **the article:** Nsiah-Frimpong *et al.*’s (2020) article on farmers’ knowledge, attitudes and practices towards management of cassava pests and diseases in what appears to be a service to Gates Foundation researchers and grant recipients, provides a robust opportunity for information sharing and creation. Given the importance of cassava to sub-Saharan Africa and Ghana in particular, this article is timely and critical for agricultural research and development.

**Background: **The article gives a rich background to the study based on current existing global and national literature in Ghana and other Sub Saharan African countries to emphasis the importance of cassava crop for both global income generation and food security. The developing argument is that cassava, is an important agro-economic activity not only in Ghana and other parts of Africa but also globally because it serves as food for 800 million people in the world (Fondong & Rey, 2018). However, it would have been helpful if the authors had clarified if KAP is a model or an analysis framework and how each of the concepts is defined, measured and operationalized in the study. The objective on finding out the preferred cassava variety traits of farmers is not clear to indicate if these are traits related to pest and disease management or genera, and if the analysis will target different gender categories.

 Notwithstanding, based on the literature, review, the authors clearly identified the gap necessitating their study.

**Key message**: The article portrays cassava crop as an exemplary crop because of its agro-ecological adaptation, in-ground storage capability and capacity to ensure all year-round food availability and its great potential for industrialization due to its high starch content and varied product utilization. The article asserts that despite the crops’ potential productivity, the efforts to bring it to the optimal level remain low; in addition limited small holder farmers’ capacity to control plant pests and diseases, access cultivars and little knowledge of the pests and diseases or management practices complicate the situation.

**Methodologically**, the research that was conducted in the two cassava growing regions of Ghana, Northern and Eastern is based on interviews, quantitative data and review of related literature. The choice of mixed methods approach is commendable and captures the multidisciplinary nature of the research team. However, while the study employed a mixed method research approach, the qualitative data collection methods and process was muted which faults the intended mixed methods approach - it is also not clear what the authors mean by a mixed method research questionnaire was adopted with both closed and open-ended questions.

 Similarly, while the quantitative data analysis is clearly explained, qualitative data analysis has some gaps which perhaps explains the absence of qualitative data. The study would have been richer had the team ensured balance between men and women cassava farmers during the selection of respondents as key to address the different segments of the target population. Whereas the authors at one moment indicate to have selected 10 cassava farmers per community and in other sections selected households, it would been better to clearly and consistently indicate the actual sampling unit and clarify the methods used to collect data.

**Results:**

**Strengths and weaknesses:** The article presents rich and evidenced-based findings statistically illustrated. Given that this is a Bill and Melinda Gates funded project-known for putting women and girls at the centre of development, (Gates (2014 ^1^), I would have expected gender being given priority which I find missing. For example, while the authors ably showed revealed that 77.7% of the respondents were males and 22.3% females, they do not proceed to give sex disaggregated data at other levels for example from the majority of the respondents (69.1%) who had some formal education, how many were females and how many were males. Perhaps this could have presented different insights regarding the differentiated knowledge, attitudes and practices. Such silences on sex disaggregation denies the readers interesting insights. Another example is the finding that 77.7% of the respondents were members of an agricultural organization, one would be interested in knowing how many were men and how many were women. Likewise, gender differences would have also fitted in the findings on differences across the agro-ecologies, age, years of cultivating cassava, years in school and household size. 

 Another missed opportunity are the voices from the qualitative findings. While the study was based on a mixed methods approach, the reader cannot put a finger on the findings generated qualitatively. Just like collecting qualitative data seems to have been by passed, qualitative findings faced the same fate. The authors refer to qualitative and quantitative methods, but the results presented clearly indicate that only the survey was conducted or qualitative data was left out. In future studies, the authors should ensure that when qualitative methods are employed, they should generate publishable data alongside the quantitative data. As Fossey *et al.* (2002 ^2^) argues that Qualitative research aims to address questions concerned with developing an understanding of the meaning and experience dimensions of humans’ lives and social worlds, the collected qualitative data would have augmented the statistical results to answer the why questions. For example, what explains 61.8% of the farmers’ perception of whiteflies ( *Bemisia tabaci *Gennadius), grasshoppers ( *Zonocerus variegatus*), aphids ( *Aphis gossypii *Glover), mealybugs ( *Phenacoccusmanihoti*), termites ( *Isoptera*), and grasscutters ( *Thryonomys swinderianus*) as the most common and damaging pests to their crops? This would have been explained by qualitative data that focuses on details and meanings research participants attach to their experiences.

**Discussion:**

 The discussion is adequate and backed up with related and relevant literature. A review of the working paper by Tufan *et al.* (2018 ^3^) could help the authors provide a grounded discussion on the variation in traits preferred by men and women farmers, and probably a compelling explanation why pest and disease resistance ranked fourth on farmers’ preferred traits.

**Conclusion and recommendations:**

 The conclusions and recommendations are adequately presented and largely drawn from the study results. The authors adequately provide necessary action to address the gaps identified. Although available literatures indicate the role of farmers in breeding systems, the recommendation on *the need for the breeding team to make farmers an integral part of the breeding process, in terms of design and setting breeding priorities, to enhance uptake* does not seem to be directly linked to the study findings.

**Appendix:** ***Comments that would strengthen the article (Suggested changes and needed clarifications on the results section):***

1. For consistency and the reader to follow; use either Guinea Savannah and Forest transition or Northern and Eastern regions. Choose one and maintain same both in tables and text.
2. Table 4: Do farmers know the pests by the specified names or they have common local names used?
3. Caption/title for Table 5 needs to be revised to make it explicit. For instance, you could say: **Farmers response (** ***or scoring depending on how data was collected*** **) to the Disease-symptom assessment statements.**
4. Table 6 should come before Table 5 since it is referred to before.
5. Need to explain the mixed infections in the following statement for easy of understanding by a non-technical person: *“* *Out of the 67 respondents who experienced cassava diseases, 74.6% reported single infections and 25.4% had mixed infections.”*
6. The relationship between cassava cropping system and cassava pests and diseases management needs to be elaborated based on the results in table 7. For instance, was there an increase or decrease in incidence with specific cropping systems or intercrops?
7. Figure 2 could be improved to show the variety types cultivated by region and sex of the farmers.
8. Table 8 on names of varieties cultivated in the study areas could have been better presented by the two agro-ecologies - Guinea savannah and forest transition region. This could also probably help explain the observed incidences.
9. Table 9 could be improved to show the distribution of farmers’ awareness by sex of farmer and consequently discuss implications based on the results in the discussion section.
10. The immediate action taken by farmers of “reported to agricultural officer” need to be explained to enhance understanding. Just reporting to an agricultural officer may not be a pest and disease management practice per se but could prompt actual action guided by the options provided by the agricultural officer.
11. Based on the results, it is not clear how the overall level of effectiveness of the pest and disease management measures as presented (Table 12) was captured, processed and analyzed.
12. Figure 3: was the source of information on crop pests and diseases the same across regions? The authors may need to disaggregate the analysis by region or sex to strengthen the discussion. The results should also be arranged in descending order to make the figure look smarter.
13. The number of tables is quite high. The authors could reduce on the number by merging some tables for instance tables 1 & 2, then 10 & 11 among others. For the tables, the authors should opt to report either frequency or percentage with sample size (n) specified in the table header or title. Further, try as much as possible to sort the frequencies or percentages in descending order (from highest to lowest) *where applicable.*

Is the work clearly and accurately presented and does it cite the current literature?

Yes

If applicable, is the statistical analysis and its interpretation appropriate?

Yes

Are all the source data underlying the results available to ensure full reproducibility?

Partly

Is the study design appropriate and is the work technically sound?

Yes

Are the conclusions drawn adequately supported by the results?

Yes

Are sufficient details of methods and analysis provided to allow replication by others?

Partly

Reviewer Expertise:

Peace Musiimenta's area of research is gender and development with a focus on gender and agricultural transformation, women's socio-economic empowerment, the costs of gender inequality in agricultural productivity and gender and education. Peace Musiimenta is an experienced gender analyst and trainer who holds a PhD in Gender studies. She has attained further professional training Post graduate for Professional Gender Trainers (PDPGT) from Royal Tropical Institute (KIT) Netherlands, a gender and qualitative research specialist for GREAT (Gender Responsive Researchers Equipped for Agricultural Transformation) course. She is a lecturer in the School of Women and Gender Studies, Makerere University  and a gender and qualitative trainer for GREAT.  Losira Nasirumbi Sanya is a highly innovative, proactive and result oriented social and development research professional, holds a PhD in Agricultural and Rural Innovation, has professional training in Integrated Agricultural Research for Development (IAR4D); designing and managing multi-stakeholder processes for rural innovation; value chains and market-oriented research; and Monitoring, Evaluation and Impact Assessment of R&D Investments in Agriculture. She is a lecturer in the College of Agriculture and Environmental Sciences  (CAES), a quantitative research specialist and GREAT (Gender responsive researchers equipped for Agricultural Transformation) trainer.  

We confirm that we have read this submission and believe that we have an appropriate level of expertise to confirm that it is of an acceptable scientific standard, however we have significant reservations, as outlined above.

## References

[1] : Putting women and girls at the center of development. *Science*.2014;345(6202) : 10.1126/science.1258882 1273-5 10.1126/science.1258882 25214610

[2] : Understanding and evaluating qualitative research. *Aust N Z J Psychiatry*.2002;36(6) : 10.1046/j.1440-1614.2002.01100.x 717-32 10.1046/j.1440-1614.2002.01100.x 12406114

[3] : State of the Knowledge for Gender in Breeding: Case Studies for Practitioners. Lima (Peru). Working Paper. No. 3. *CGIAR Gender and Breeding Initiative*.2018; Reference source

# Response

**Published:** 2021-02-02

**Authors:** BNF Frimpong

**Affiliations:** CSIR-Crops Research Institute, Ghana

Dear Reviewers,

 The authors appreciate your time, constructive criticisms and suggestions to enrich the article. We hope to clarify some of the issues raised as follows:

 1. The KAP is already clarified in the background as a model and the operationalization of the KAP concepts were also done under data analysis. Under the data analysis section, how effectiveness was measured was also explained. 

 2. The objective of finding out preferred traits by farmers was to ascertain whether "pest and disease resistance" was deemed as an important trait by farmers since farmers will allocate resources to issues that are relevant to them.

 3. Under the data collection section, the authors never stated they employed mixed method approach to data collection but a mixed questionnaire to capture both open and closed ended questions. This was a survey and mixed research questionnaire was used as an instrument to elicit both qualitative and quantitative information.

 4. Again, the unit of analysis is explicit as individual cassava farmers which could be referred to under the data collection section.

 5. Most of the remaining questions are centered on the gender dimension of the study may be because of the reviewers research interests. The gender perspective could be looked at holistically in our next study as it was not the main focus of this study. This is a small aspect of the WAVE project and this could be looked at in subsequent papers. 

 6. Suggestions on table merging and caption rephrasing have however been effected accordingly in the new version.

 7. The underlying data was deposited in the Harvard Dataverse repository and the DOI is indicated in the article for reference and reproducibility.

# Peer review: Reviewer response for version 1

**DOI:** 10.21956/gatesopenres.14290.r29065

**Published:** 2020-07-23

**Authors:** Andrew Sarkodie Appiah

**Affiliations:** 1 Biotechnology Centre, Biotechnology and Nuclear Agricultural Research Institute (BNARI), Accra, Ghana

**Abstract:**

- The abstract adequately summarizes the objectives, methodology and major findings. It is however, suggested that the authors should modify the sentence ‘ *Some of the farmers observed mixed infections on their farms*’ to read ‘ *Some of the observed multiple symptoms on their farms’* since no definite tests were carried out to ascertain mixed infections.

**Introduction:**

- The introduction adequately provides background to the research, clearly states the problem, justification and objectives of the study. However, the authors are to do the following: 'African cassava mosaic virus' should be written in italics as ' *African cassava mosaic virus' *since it is a standard virus name.

**Methodology:**

- The methodology adopted was consistent with the objective of the study.

 

**Results and Discussion:**

- The results are well presented and the discussion highlights the importance and relevance of the results.

 

**Conclusions:**

- The conclusions drawn are adequately supported by the results.

Is the work clearly and accurately presented and does it cite the current literature?

Yes

If applicable, is the statistical analysis and its interpretation appropriate?

Yes

Are all the source data underlying the results available to ensure full reproducibility?

No source data required

Is the study design appropriate and is the work technically sound?

Yes

Are the conclusions drawn adequately supported by the results?

Yes

Are sufficient details of methods and analysis provided to allow replication by others?

Yes

Reviewer Expertise:

Plant Virology, Plant viral disease epidemiology, Diagnostics, Pathogen diversity analysis, Sequencing, Vector transmission.

I confirm that I have read this submission and believe that I have an appropriate level of expertise to confirm that it is of an acceptable scientific standard.

# Response

**Published:** 2021-02-01

**Authors:** BNF Frimpong

**Affiliations:** CSIR-Crops Research Institute, Ghana

Thank you Dr. Sarkodie Appiah for the constructive review. All suggestions have been effected accordingly in the new version.

 The underlying data was deposited in the Harvard Dataverse repository and the DOI is indicated in the article for reference.

 Benedicta Nsiah Frimpong (Author).
